# Prevalence of *Acinetobacter baumannii* bacteremia in intensive care units of Ibn Rochd University Hospital, Casablanca

**Published:** 2017-12

**Authors:** Assiya El Kettani, Fakhreddine Maaloum, Idrissa Diawara, Khalid Katfy, Nadia Harrar, Khalid Zerouali, Houria Belabbes, Naima Elmdaghri

**Affiliations:** 1 Department of Microbiology, Faculty of Medicine and Pharmacy, Hassan II University of Casablanca, Morocco; 2 Bacteriology-Virology and Hospital Hygiene Laboratory, University Hospital Centre Ibn Rochd of Casablanca, Morocco

**Keywords:** *Acinetobacter baumannii*, Bacteremi, Antibiotic resistance, *bla*_OXA-51_, *bla*_OXA-23_

## Abstract

**Background and Objectives:**

*Acinetobacter baumannii* bacteremia are grave because of the multi-resistance of the organism to antibiotics. This study aimed to determine the prevalence of *A. baumannii* isolated from blood cultures and to describe their antibiotic resistance patterns.

**Materials and Methods:**

A retrospective longitudinal study was conducted on blood cultures between 2010 and 2014 from all Ibn Rochd University Hospital intensive care units; it was based on the exploitation of microbiology laboratory database (duplicates were excluded). Isolation and identification of *A. baumannii* were performed according to standard techniques of bacteriology and susceptibility testing as recommended by the CLSI. PCR was used to detect β-Lactamase genes, *bla*_OXA-51_, *bla*_OXA-23_.

**Results:**

Among the 4232 samples received at the laboratory, 2402 (56.8%) were positive. Negative coagulase *Staphylococcus* was isolated in 21.6% of cases followed by *A. baumannii* (9.2%), and *K. pneumoniae* (9.1%). *A. baumannii* strains were resistant to most antibiotics tested: imipenem (75.7%), ceftazidim (85.4%), cefotaxim (98.6%), gentamicin (78.1%), amikacin (63.5%) and ciprofloxacin (88.2%). All *A. baumannii* strains, resistant to carbapenem, tested were positive for *bla*_OXA-51_ genes and 87.5% expressed the *bla*_OXA-23_ genes.

**Conclusion:**

*A. baumannii* was the second germ frequently isolated from blood cultures in intensive care units. It was multi-resistant to antibiotics. The strengthening of hospital hygiene measures and surveillance of antibiotic resistance is needed to limit the spread of germs and to optimize the management of antibiotics.

## INTRODUCTION

*Acinetobacter baumannii* is a Gram-negative pathogen. It is frequently associated with nosocomial infections (bacteremia, pneumonia, meningitis and urinary tract infections). It was also recognized worldwide as an emerging cause of nosocomial outbreaks and listed by the American Society of Infectious Diseases (IDSA) as one of the six most hazardous microorganisms (1). *A. baumannii* bacteremia in intensive care units (ICUs) are responsible of a significant morbidity and mortality and create a therapeutic problem due to the multidrug resistance of the organism to antibiotics (2, 3). *A. baumannii* have a natural resistance to antibiotics, but also an acquired resistance by production of enzymes: the β-lactamases and especially metallo-β-lactamase and oxacillinases and by efflux pumps and changing porins (4). Carbapenem hydrolyzing β-lactamases belonging to oxacilinases enzymes are the main enzymes contributing to the inactivation of imipenem in *A. baumannii* (5, 6).

The aim of this study was to determine the prevalence of *A. baumannii* isolated from blood cultures performed in intensive care units of Ibn Rochd University hospital at Casablanca-Morocco during the last five years (2010–2014) and to describe the evolution of the organism’s resistance to antibiotics. Then, to detect the presence of Oxacillinases: OXA-23 and OXA-51 among the isolates showing resistance to imipenem.

## MATERIALS AND METHODS

### Samples collection (Patients samples).

A retrospective longitudinal study was carried out on blood cultures from all ICUs in Ibn Rochd University Hospital between 2010 and 2014. It was based on the exploitation of the Microbiology laboratory database (duplicates of the same patient were excluded). All bacteria including *A. baumannii* identified by the standard bacteriological methods in the routine diagnosis of bacteremia from all ICUs were examined. PCR was used to target the *bla*_OXA-23_ and *bla*_OXA-51_ among the imipenem resistant isolates *A. baumannii*.

### Antimicrobial susceptibility testing.

Antimicrobial susceptibility testing was done for all *A. baumannii*. Disk diffusion test was used to determine the susceptibity of isolates to ceftazidim (30 μg), 30 μg cefotaxim (30 μg), 30 μg amikacin (30 μg), 10 μg gentamicin (10 μg), ciprofloxacin (5 μg), ampicillin/sulbactam (10 μg), piperacillin/tazobactam (85 μg), netilmicin (10 μg), Tobramycin (10 μg), trimethoprim/sulfametoxazol (25 μg), tetracycline (30 μg), imipenem (10 μg). E-test was used to determine the MICs for colistin (Biomérieux Marcy l’Etoile France). *E. coli* ATCC 25922 was used as a quality control strain. Results were interpreted according to the breakpoints recommended by the Clinical and Laboratory Standards Institute (CLSI, 2014).

### Preparation of DNA and PCR.

All *A. baumannii* resistant to imipenem were grown on Mueller–Hinton (MH) agar plates (Bio-Rad, Marnes-la-Coquette, France) for 18–24 hours at 37°C, bacterial cells were suspended in 500 μl of ultrapure water. Suspension was heated at 100°C for 10 min and immediately frozen at 0°C for 5 min. 300 μl of supernatant were then recovered after centrifugation of 14000 g for 10 min. Supernatant containing DNA was stored at −20°C until further use (5).

PCR protocol described by Sandle et al. (6) was used for detection of *bla*_OXA-23_ and *bla*_OXA-51_ among the imipenem resisrant isolates.

### Statistical analysis.

Data were analyzed with Epi-Info 7 (Centers for Disease Control, Atlanta, Georgia, USA) and Microsoft Excel. The chi square test or Fisher’s exact test was performed to compare proportions. Differences were considered significant if the p-value is <0.05.

## RESULTS

**Fig. 1.**
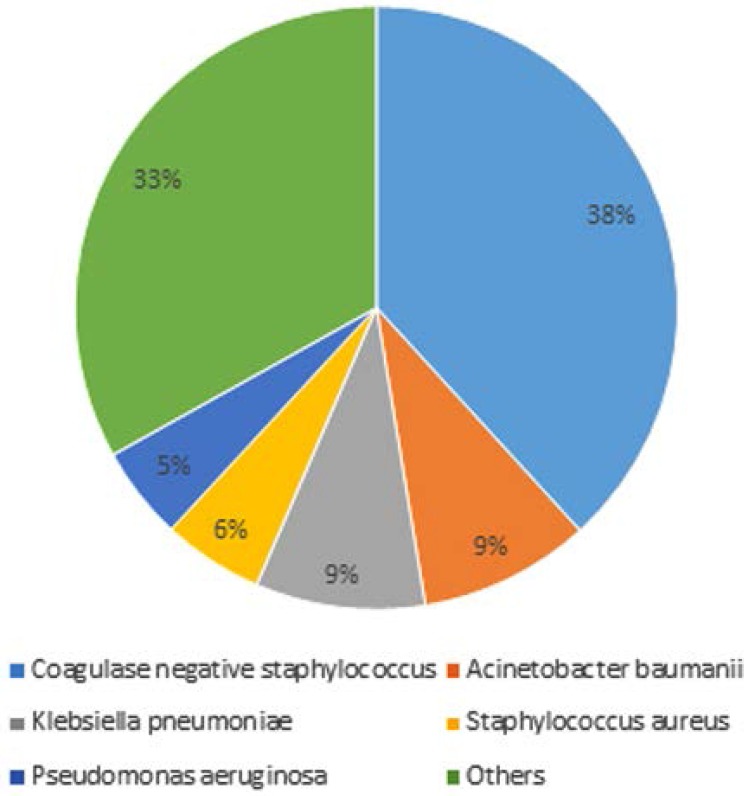
Distribution of bacteria isolated from intensive care units blood cultures

During the study period, a total of 4232 non-duplicate blood cultures from 4232 patients were received at the laboratory. Of these, 2402 blood cultures (56.8%) were positive. Negative coagulase Staphylococcus was isolated in 21.6% of cases followed by *A. baumannii* (9.2%) and *K. pneumoniae* (9.1%) (Fig. 1).

The prevalence of *A. baumannii* strains increased from 7% in 2010 to 10% in 2011, 2012 and 2013, and then decreased slightly in 2014 (9%).

**Fig. 2.**
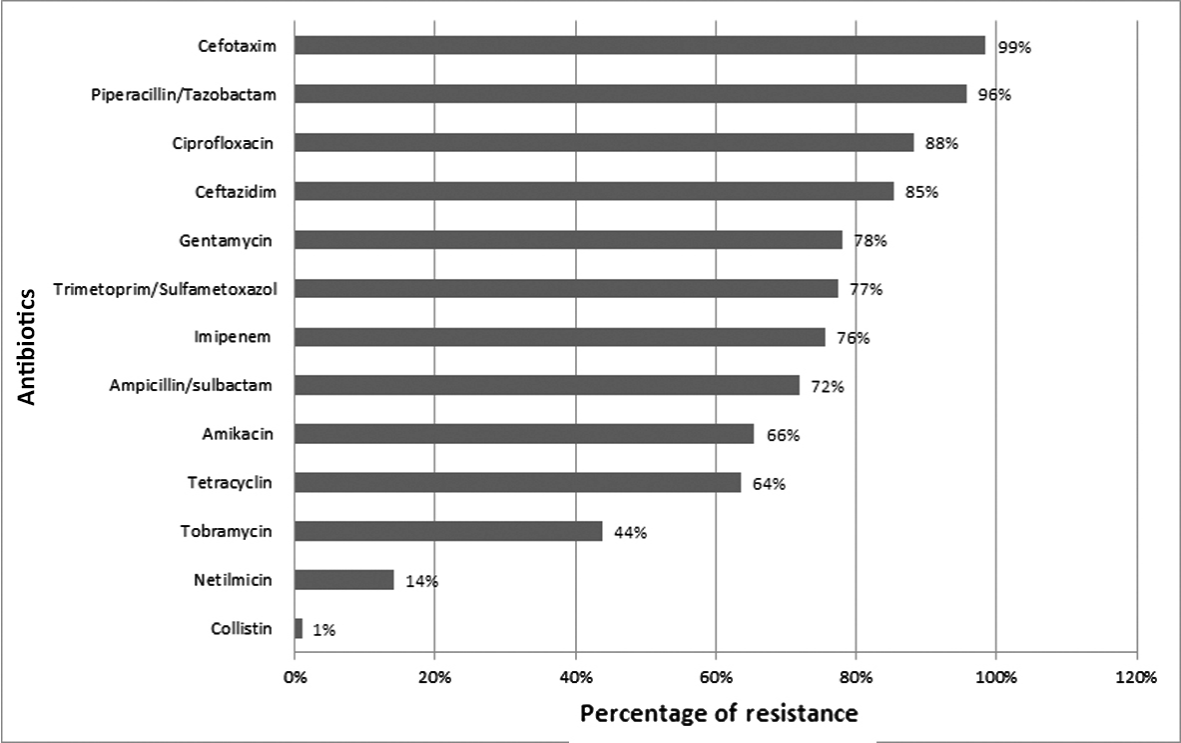
Antibiotic resistance of *A. baumannii* in Intensive care units

*A. baumannii* strains were resistant to: imipenem (75.7%), ceftazidime (85.4%), cefotaxime (98.6%), gentamicin (78.1%), ciprofloxacin (88.2 %), the netilmicin (14%) and colistin (1%) (Fig. 2).

**Fig. 3.**
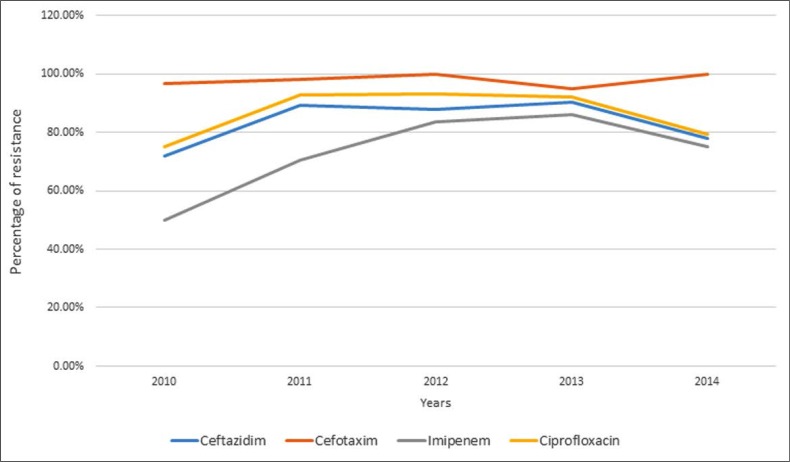
Evolution of *A. baumannii* resistance to antibiotics during the study period

The evolution of antibiotic resistance was determined for ceftazidim, cefotaxim, imipenem and ciprofloxacin. It was relatively stable for the third generation cephalosporins and fluoroquinolones. However, it increased for imipenem, from 50% in 2010 to 86% in 2013 (*p*=0.0003) and then decreased slightly in 2014 (75%) (*p*=0.35) (Fig. 3).

**Fig. 4.**
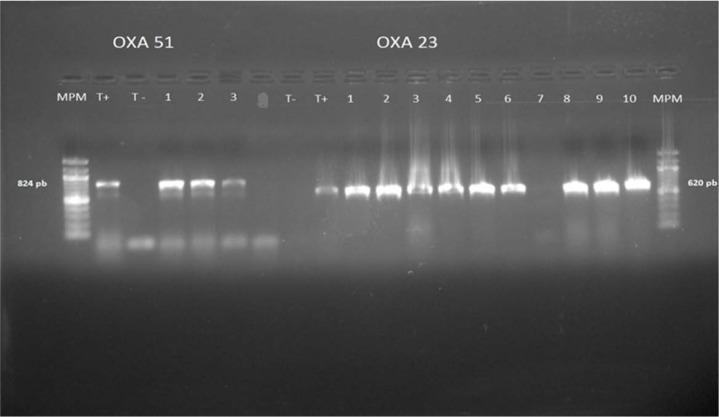
PCR for *bla*_OXA-51_ and *bla*_OXA-23_ genes of *A. baumannii* isolated from ICU blood cultures

As for the genotypic characterization of *A. baumannii* strains resistant to imipenem, determined by PCR, all *A. baumannii* strains were positive for *bla*_OXA-51_ genes and 87.5% (n=147) expressed the *bla*_OXA-23_ genes (Fig. 4).

## DISCUSSION

In our study, *A. baumannii* was the second germ frequently isolated from blood cultures in ICUs between 2010 and 2014 (9.2%). It was characterized by a multi-resistance to the antibiotics tested (C3G, aminoglycosides, fluoroquinonolones and imipenem). It was however, sensitive to colistin in 99% of cases.

The evolution of antibiotic resistance during the study period was increasing especially for imipenem (from 50% in 2010 to 75% in 2014). All isolates of *A. baumannii*, resistant to carbapenem, were positive for *bla*_OXA-51_ genes and 87.5% were positive for *bla*_OXA-23_.

*A. baumannii* is considered as an opportunistic pathogen that can survive in austere conditions. It is responsible of an increasing rate of severe nosocomial infections. They affect especially immunocompromised patients, exposed to prolonged stays in ICUs and having a previous exposure to antibiotics; carbapenems and 3^rd^ generation cephalosporins are the most involved, followed by fluoroquinolones, aminoglyco-sides and metronidazole (2, 7). Other factors that are associated with the occurrence of *A. baumannii* bacteremia are: assisted ventilation, central catheterization, urinary catheters, and nasogastric probes (8).

The distribution of bacteria in our study is consistent with a previous study in the same hospital in which *A. baumannii* represented 13% of the bacteria isolated from blood cultures (9). It should be noted that the negative coagulase staphylococci are contaminants in the majority of cases and only 10% to 30% were clinically significant (10). Nevertheless, this distribution contrasts with data from a French study where *S. aureus* (18.4%), *E. coli* (15.4%) and *S. epidermidis* (14.4%) accounted for almost a half (48.3%) of blood cultures isolated microorganisms; *Candida albicans* represented 2.5%. *A. baumannii* was rarely isolated (0.6% of microorganisms responsible of nosocomial infections in all sites) (11).

Resistance to the 3^rd^ generation cephalosporins and ciprofloxacin, in Algeria, Tunisia and France, is over than 50% (11–14) as it was in our study.

Resistance to imipenem (treatment of choice for *A. baumannii* infections) is variable: 3.3% in France, 60% to 89.9% in Tunisia and 48% in Algeria (11–14). Our study objectified an increased resistance from 50% in 2010 to 86% in 2013 with a slight decrease in 2014 (75%). This could be due to pressure selection exerted by excessive prescription of imipenem.

The acquired carbapenem resistance in *A. baumannii* is often associated with carbapenemase production; IMP, VIM and SIM-type metallo-β-lactamase production or the OXA-24, OXA-23 and OXA-58 type class D carbapenemases. But also with the over production of natural oxacillinase (OXA-51) (15). OXA-23 and OXA-51 are considered as the most prevalent among *A. baumannii* carbapenem resistance (16). In our study, all *A. baumannii* strains were positive for *bla*_OXA-51_ genes and 87.5% (n=147) for *bla*_OXA-23_.

Strains of *A. baumannii* harbouring OXA-23 enzymes have been identified in Brazil, Argentina and Colombia (17–21). Moreover, carbapenemases belonging to OXA-23 subgroup have been detected in Europe, Australia, Tahiti, China, Korea, Singapore, Vietnam, USA, Libya and Pakistan (22) Mobilization of the *bla*_OXA_ genes is determined by the presence of insertion sequences and transposons, and therefore has a high potential to spread (23). That is why it is essential to conduct molecular genotyping studies as well as characterizing the carbapenemases found in specific geographic areas, to highlight molecular evolution of both genes and resistant clones.

The multi-drug resistance of this germ has led to a renewed interest in colistin, to the association of antibiotics (rifampicin-colistin, colistin-imipenem) and the use of new antibiotics such as tigecycline to overcome therapeutic impasses (24).

## CONCLUSION

*A. baumannii* was the second germ that was frequently isolated in blood cultures of intensive care units in the last five years in Ibn Rochd University Hospital. It was multi-resistant to antibiotics with a particular increasing resistance to imipenem. The strengthening of hospital hygiene measures and antibiotic resistance surveillance is needed to limit the spread of multi resistant bacteria and to optimize the management of antibiotic therapy.
